# Cerebellar Globular Cells Receive Monoaminergic Excitation and Monosynaptic Inhibition from Purkinje Cells

**DOI:** 10.1371/journal.pone.0029663

**Published:** 2012-01-03

**Authors:** Moritoshi Hirono, Fumihito Saitow, Moeko Kudo, Hidenori Suzuki, Yuchio Yanagawa, Masahisa Yamada, Soichi Nagao, Shiro Konishi, Kunihiko Obata

**Affiliations:** 1 Obata Research Unit, RIKEN Brain Science Institute, Saitama, Japan; 2 Yamada Research Unit, RIKEN Brain Science Institute, Saitama, Japan; 3 Laboratory for Motor Learning Control, RIKEN Brain Science Institute, Saitama, Japan; 4 Department of Pharmacology, Nippon Medical School, Tokyo, Japan; 5 Japan Science and Technology Agency, CREST, Tokyo, Japan; 6 Department of Genetic and Behavioral Neuroscience, Gunma University, Graduate School of Medicine, Gunma, Japan; 7 Department of Neurophysiology, Kagawa School of Pharmaceutical Sciences, Tokushima Bunri University, Kagawa, Japan; Tokyo Medical and Dental University, Japan

## Abstract

Inhibitory interneurons in the cerebellar granular layer are more heterogeneous than traditionally depicted. In contrast to Golgi cells, which are ubiquitously distributed in the granular layer, small fusiform Lugaro cells and globular cells are located underneath the Purkinje cell layer and small in number. Globular cells have not been characterized physiologically. Here, using cerebellar slices obtained from a strain of gene-manipulated mice expressing GFP specifically in GABAergic neurons, we morphologically identified globular cells, and compared their synaptic activity and monoaminergic influence of their electrical activity with those of small Golgi cells and small fusiform Lugaro cells. Globular cells were characterized by prominent IPSCs together with monosynaptic inputs from the axon collaterals of Purkinje cells, whereas small Golgi cells or small fusiform Lugaro cells displayed fewer and smaller spontaneous IPSCs. Globular cells were silent at rest and fired spike discharges in response to application of either serotonin (5-HT) or noradrenaline. The two monoamines also facilitated small Golgi cell firing, but only 5-HT elicited firing in small fusiform Lugaro cells. Furthermore, globular cells likely received excitatory monosynaptic inputs through mossy fibers. Because globular cells project their axons long in the transversal direction, the neuronal circuit that includes interplay between Purkinje cells and globular cells could regulate Purkinje cell activity in different microzones under the influence of monoamines and mossy fiber inputs, suggesting that globular cells likely play a unique modulatory role in cerebellar motor control.

## Introduction

Characterization of individual cellular elements and their synaptic connections in the cerebellum is important for precise understanding of the mechanisms of motor coordination. The cerebellar cortex has been assumed to possess a low degree of variability in its interneuron types compared to other brain regions such as the hippocampus and cerebral cortex [1]–[3]. Recent studies, however, revealed that cerebellar cortical interneurons are far more diverse and heterogeneous than traditional classifications acknowledge [4]–[7]. In the cerebellar granular layer, two types of large-sized inhibitory interneurons, Golgi and Lugaro cells, are distributed [8]–[11]. Golgi cells, which are distributed ubiquitously throughout the granular layer, have large polygonal soma with radial dendrites, and constitute a major group of glycinergic/GABAergic interneurons [12]–[14]. Meanwhile, Lugaro cells are located in the upper granular layer and smaller in number, and possess bidirectional dendrites spreading along the Purkinje cell layer [9]–[11], [15], [16]. Lugaro cells in the rat cerebellum are characterized with robust firing following the activation of serotonin (5-HT) receptors, leading to the inhibition of molecular layer interneurons: basket cells and stellate cells, as well as Golgi cells, whereas 5-HT does not induce firing in rat Golgi cells [17]–[19]. In the upper granular layer, three types of smaller-sized inhibitory interneurons are identified on the basis of morphological criteria [5]–[7]: small Golgi cells, small fusiform Lugaro cells, and globular cells. Small Golgi cells and small fusiform Lugaro cells are likely to possess the same physiological properties as large-sized Golgi cells and Lugaro cells, respectively [6], [10], [11]. The physiological property of globular cells, however, has never been reported, because globular cells are small in number, and intermingled with small Golgi cells. In the present study, using GAD67^+/GFP^ mice that express GFP specifically in GABAergic neurons [20], we located these interneurons *in situ*, and characterized the properties of small Golgi cells, small fusiform Lugaro cells, and globular cells by electrophysiological, pharmacological and morphological methods. We found that globular cells are directly and robustly inhibited by Purkinje cell axon collaterals, and excited by mossy-fiber and monoaminergic inputs, suggesting that globular cells constitute a new category of cerebellar cortical interneurons which integrate the Purkinje cell inhibitory inputs with the excitatory mossy fiber inputs under the monoaminergic modulation.

## Results

### Morphological and firing properties of small inhibitory interneurons

**Figure 1:**
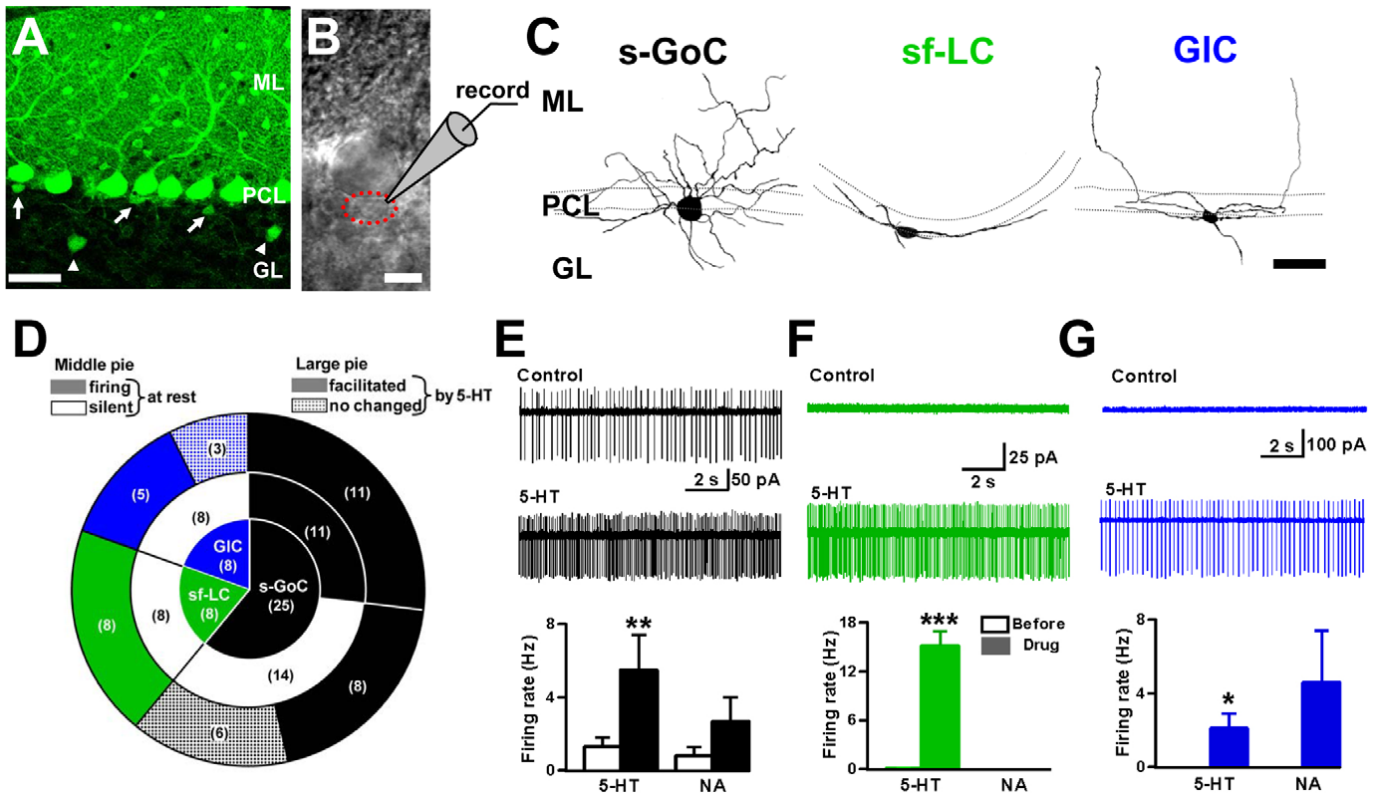
Morphology and monoaminergic modulation of small inhibitory interneurons in the cerebellar granular layer. (*A*) Confocal image of the cerebellar cortex in GAD67^+/GFP^ mouse. GL: granular layer, PCL: Purkinje cell layer, ML: molecular layer. GFP fluorescence shows GABAergic neurons. Arrows indicate small inhibitory interneurons underneath the PCL. Arrowheads point to Golgi cells (GoCs) in the middle of GL. Scale bar, 50 µm. (*B*) Transmitted light image of a soma of a globular cell (GlC) (dotted red circle) located close to a PC body. Scale bar, 10 µm. (*C*) Alexa Fluor 594 fluorescent images of the three types of interneurons beneath the PCL. Scale bar, 50 µm. (left) Typical small GoC (s-GoC), having a polygonal and globular soma and extending dendrites radially. (middle) Typical small fusiform Lugaro cell (sf-LC), having a fusiform soma and extending dendrites to both sides of the soma. (right) Typical GlC, having a small globular soma and extending dendrites mostly in the PCL. (*D*) Pie diagram showing the distribution of cell types tested. In parentheses, the number of cells tested. The small pie diagram indicates the populations of the tested three types of interneurons. The intermediate pie diagram indicates the numbers of cells firing or silent at rest. The large pie diagram indicates the number of cells whose firing was facilitated by 5-HT or not. (*E*) for an s-GoC. Upper trace, specimen record of spontaneous spikes at rest. Lower trace, under 5-HT perfusion. Histogram at the bottom, spike discharge rates under 5-HT or NA. (*F*) Similar to (E) but for an sf-LC. (*G*) for a GlC.

To visualize small GABAergic interneurons in the granular layer, we used sagittal cerebellar slices from GAD67^+/GFP^ mice (Fig. 1*A*) [20]. Lugaro cells in the rat cerebellum have been shown to be silent and to become intensively active following 5-HT application [17], [18]. Thus, we first observed spontaneous firing of small GABAergic interneurons located just beneath the Purkinje cell somata (within 10 µm from the lower border of the Purkinje cell layer) using loose cell-attached recordings. We measured the firing rate of 41 randomly targeted GFP-positive interneurons and examined the effects of monoamines, 5-HT and noradrenaline (NA). Next, the same recorded neurons were labeled by a fluorescent dye, Alexa Fluor 594, injected from a second electrode in the whole-cell recording mode, which enabled the characterization of synaptic inputs with morphology of recorded neurons. Twenty-five of 41 cells tested had a polygonal soma with a number of radial dendrites extending in both the granular layer and the molecular layer (averaged processes from soma, 6.5±0.4, *n* = 14; Fig. 1*C* and Table 1), showing some of the morphological properties of Golgi cells. We refer to such Golgi cells as “small Golgi cells”, because their membrane capacitance was significantly smaller (19.8±1.4 pF, *n* = 25) than that of Golgi cells, which were recorded in the middle of the granular layer (32.8±3.8 pF, unpaired Student's *t*-test, P<0.001, *n* = 9). Approximately half of them showed spontaneous firing at rest (2.9±1.0 Hz, 11 of 25 cells), and even at a physiological temperature 4 of 10 small Golgi cells tested remained silent. The firing rates of small Golgi cells were increased by 10 µM 5-HT (5.5±1.9 Hz, P<0.01, *n* = 25) (Fig. 1*D* and *E*). Another monoamine, NA (10 µM), increased the firing rate in 7 of 11 small Golgi cells tested (0.8±0.5 to 2.7±1.3 Hz, P = 0.07, n = 11; Fig. 1*E*). Eight of the 41 cells had a Lugaro cell-like fusiform soma with dendrites laterally extending from both sides of the soma (3.1±0.1 processes, *n* = 10; Fig. 1*C* and Table 1). The mean cell capacitance (13.7±3.1 pF, *n* = 8) was smaller than that of the typical Lugaro cells located in the middle of the granular layer (18.1±0.8 pF, unpaired Student's *t*-test, P<0.05, *n* = 5). We termed them “small fusiform Lugaro cells”. In cell-attached mode, small fusiform Lugaro cells did not fire at rest (except for one cell firing at <0.5 Hz), and 5-HT increased the firing rates of small fusiform Lugaro cells markedly (from 0.06±0.06 to 15.2±1.7 Hz, P<0.001, *n* = 8) (Fig. 1*D* and *F*). By contrast, NA induced no spike discharge in small fusiform Lugaro cells (n = 4; Fig. 1*F*). We observed another-type of small GABAergic interneurons which were different from either small Golgi cells or small fusiform Lugaro cells. These interneurons (*n* = 8) had a small globular soma with several processes extending mostly along the PC layer (4.8±0.1 processes, *n* = 26; Fig. 1*C* and Table 1) and a few processes going into the molecular layer. On the basis of these morphological characteristics, we termed them “globular cells” [5]–[7] (see Discussion). Globular cells did not fire at rest (*n* = 8), and 5-HT induced firing in 5 of 8 cells (from 0.0±0.0 to 2.1±0.8 Hz, P<0.05, *n* = 8; Fig. 1*D* and *G*), although the magnitude of firing facilitation was smaller than that of small fusiform Lugaro cells (Mann-Whitney *U* test, P<0.001). Five of 6 globular cells showed a tendency of firing facilitation by NA (from 0.0±0.0 to 4.6±2.8 Hz, P = 0.16, *n* = 6; Fig. 1*G*). Notably, two silent globular cells induced robust firing in response to NA; to the maximum firing rates of 12.7 and 14.0 Hz. There is a significant difference in the NA-induced firing effect between small fusiform Lugaro cells and globular cells (Mann-Whitney *U* test, P<0.05). These results suggest that small inhibitory interneurons which show spontaneous firing at rest are Golgi cells, and sensitivity to 5-HT is not sufficient for clearly classifying these small interneurons.

**Table 1:** Morphological properties of small inhibitory interneurons.

	s-GoC	sf-LC	GlC	
No. of primary processes from soma	6.5±0.4	3.1±0.1	4.8±0.1	s-GoC>GlC>sf-LC
No. of branch points (≤50 µm from soma)	6.0±0.5	0.50±0.17	0.75±0.18	s-GoC>sf-LC≈GlC
No. of primary processes entering the ML	3.0±0.1	0.70±0.20	1.3±0.2	s-GoC>sf-LC≈GlC
Size (µm^2^)	174±15	119±9	79±4	s-GoC>sf-LC>GlC
GA/SA	1.09±0.02	1.83±0.11	1.38±0.04	s-GoC<GlC<sf-LC
Length of initial branch points from soma (µm)	18.4±1.0	24.0±2.0	23.2±2.6	s-GoC≈sf-LC≈GlC

S-GoC: small Golgi cell; sf-LC: small fusiform Lugaro cell; GlC: globular cell. Morphological data were taken from the best preserved small inhibitory interneurons infused with fluorescent dye (s-GoC: *n* = 14, sf-LC: *n* = 10, GlC: *n* = 26). The deformation was estimated by GA/SA. GA: great axis of soma (µm), SA: small axis of soma (µm). Statistical significance was examined using one-way ANOVA with Tukey's post test. > or < indicates statistically significant differences; ≈ indicates no significant difference.

### Properties of IPSCs in small inhibitory interneurons

**Figure 2:**
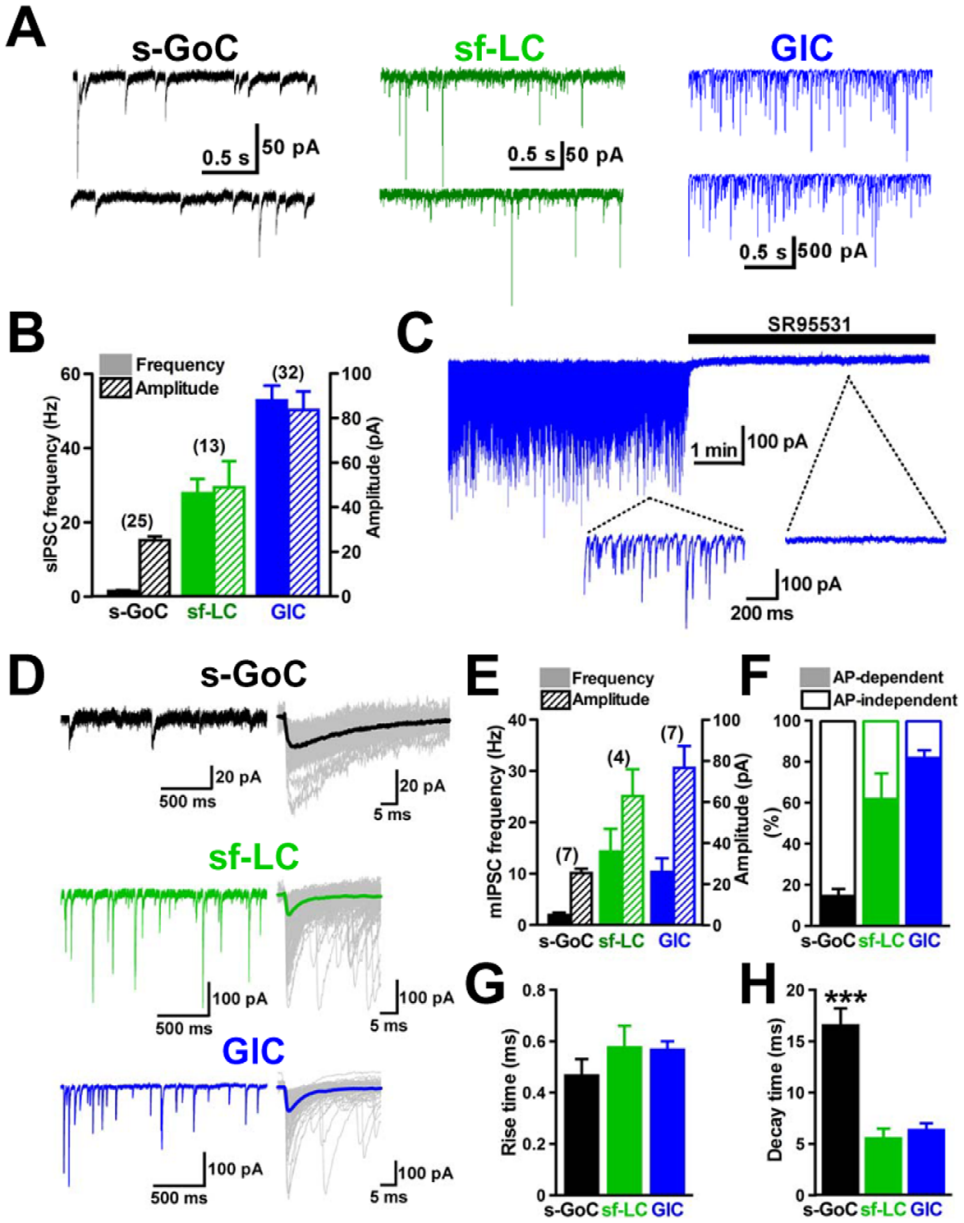
IPSCs recorded from three types of small inhibitory interneurons underneath the Purkinje cell layer. (*A*) Representative sIPSCs. (left) a small Golgi cell (s-GoC), (middle) a small fusiform Lugaro cell (sf-LC), (right) a globular cell (GlC). Neurons were voltage-clamped at −70 mV in the presence of 1 mM kynurenic acid (or 10 µM NBQX and 30 µM APV). (*B*) Averaged frequency and amplitude of sIPSCs recorded from the three types of small interneurons. (*C*) Traces of sIPSCs in a GlC. Horizontal bar indicates perfusion of a GABA_A_ receptor antagonist, SR95531. (*D*) Miniature IPSCs recorded in a slow sweep (left half). Fast sweep traces of mIPSCs (gray) are superimposed (right half), and thick lines show averaged traces. These current traces were observed in an s-GoC, sf-LC, and GlC, as indicated. (*E*) Histogram for comparing averaged frequencies and amplitudes of mIPSCs. (*F*) Histogram showing frequencies of occurrence of TTX-sensitive sIPSC. (*G* and *H*) Rise and decay phases of mIPSC traces were fitted by a single exponential function, respectively. Pooled mean values for the rise (*G*) and decay (*H*) time constants of mIPSCs in s-GoCs (*n* = 7), sf-LCs (*n* = 4), and GlCs (*n* = 7) are shown. *** P<0.001, one-way ANOVA with Tukey's post test.

We further examined the characteristics of the small interneurons. The frequency and amplitude of spontaneous IPSCs (sIPSCs) recorded from small Golgi cells were 1.5±0.2 Hz and 25.1±1.8 pA, respectively (*n* = 25; Fig. 2*A* left and *B*), while those recorded from small fusiform Lugaro cells showed a higher frequency (29.3±3.9 Hz) and larger size (48.6±10.6 pA) (*n* = 13; Fig. 2*A* middle and *B*). By contrast, the frequency and amplitude of sIPSCs recorded from globular cells were much more frequent (53.1±4.3 Hz) and larger (84.8±8.3 pA) (*n* = 32; Fig. 2*A* right and *B*). To characterize such highly frequent and phasic inhibition onto globular cells, we examined the effects of the GABA_A_ receptor antagonist SR95531 (20 µM). SR95531 completely blocked sIPSCs recorded from globular cells (Fig. 2*C*), indicating that the sIPSC in globular cells was GABAergic.

We recorded miniature IPSCs (mIPSCs) from the three types of interneurons under the suppression of action potentials by tetrodotoxin (TTX, 0.5 µM). In small Golgi cells, TTX treatment only slightly reduced sIPSC frequency (Fig. 2*F*), and the mean frequency and amplitude of mIPSCs were 1.9±0.5 Hz and 25.4±2.1 pA, respectively (*n* = 7; Fig. 2*D* top and *E*). In small fusiform Lugaro cells, TTX partially reduced sIPSC frequency (Fig. 2*F*), and the mean frequency and amplitude of mIPSCs were 14.3±4.4 Hz and 62.9±13.0 pA, respectively (*n* = 4; Fig. *2D* middle and *E*). By contrast, in globular cells, TTX strongly reduced the frequency of sIPSCs to 18±3% of the control (*n* = 7; Fig. 2*F*), and the mean frequency and amplitude of mIPSCs were 10.3±2.7 Hz and 76.6±10.7 pA, respectively (*n* = 7; Fig. 2*D* bottom and *E*). Thus, it appears that most sIPSCs recorded in globular cells were dependent on presynaptic action potentials, whereas those in small Golgi cells were not. We further compared the kinetics of mIPSCs among the three types of small interneurons (Fig. 2*G* and *H*). In small Golgi cells, the mean rise time of mIPSCs was 0.47±0.06 ms, and their mean decay time was 16.5±1.7 ms, which is significantly longer than those of small fusiform Lugaro cells and globular cells shown below (P<0.001, *n* = 7). In small fusiform Lugaro cells, the mean rise time and decay time of mIPSCs were 0.58±0.08 ms and 5.5±1.0 ms, respectively (*n* = 4). In globular cells, the mean rise time and decay time of mIPSCs were 0.57±0.03 ms and 6.3±0.7 ms, respectively (*n* = 7). Because α3-subunit of GABA_A_ receptors are selectively expressed by Golgi cells in the cerebellar cortex [21], [22], the slow decay of mIPSCs in small Golgi cells is likely attributed to the action of α3-subunit [23].

### Membrane properties of small inhibitory interneurons

**Figure 3:**
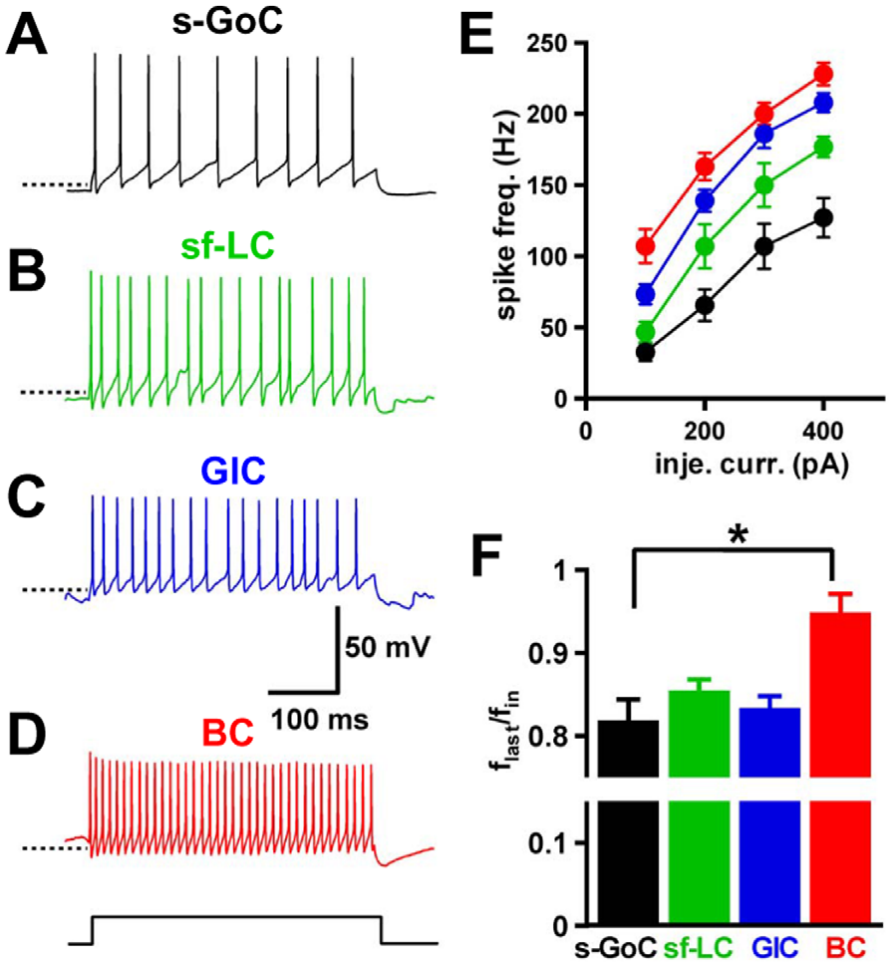
Firing properties of inhibitory interneurons located close to the Purkinje cell layer. (*A–D*), Current-clamp recordings were performed to examine firing in response to an injection current step (+100 pA, 400 ms-duration, bottom) of small Golgi cell (s-GoC, *A*), small fusiform Lugaro cell (sf-LC, *B*), globular cell (GlC, *C*), and basket cell (BC, *D*). Dot lines indicate −55 mV. (*E*) Mean firing frequencies in response to injected currents of different amplitudes in s-GoCs (black circles, *n* = 10−12), sf-LC (green circles, *n* = 6−7), GlCs (blue circles, *n* = 4−6), and BC (red circles, *n* = 6−7). (*F*) Spike frequency adaptation in response to an injected current (+200 pA, 400 ms-duration). s-GoCs (*n* = 12), sf-LC (*n* = 7), and GlCs (*n* = 6) showed accommodation, but BCs did not (*n* = 6). The firing frequency adaptation was calculated as the ratio (flast/fin) of the instantaneous frequency at the fourth and last spike intervals in a spike train. *P<0.05, one-way ANOVA with Tukey's post test.

We examined the membrane and firing properties of small inhibitory interneurons located close to the Purkinje cell layer with whole-cell current-clamp recordings. Globular cells fired at higher frequency than small Golgi cells in response to current injection (e.g. +100 pA, 400 ms-duration injection: globular cells: 68.8±6.8 Hz, n = 6, vs small Golgi cells: 32.2±4.9 Hz, n = 10; unpaired Student's *t*-test, P<0.05; Fig. 3*A, C*, and *E*). Small fusiform Lugaro cells fired at relatively lower frequency than globular cells (Fig. 3*B, C*, and *E*). Globular cells exhibited slight accommodation during firing caused by current injections similar to small fusiform Lugaro cells and small Golgi cells (Fig. 3*F*) [13], [14], [17], [24], [25]. Globular cells induced sag potential with larger amplitude during hyperpolarizing current injection (−100 pA, 400 ms-duration) compared to small Golgi cells and small fusiform Lugaro cells (Table 2) [26].

**Table 2:** Electrophysiological properties of small inhibitory interneurons.

	s-GoC	sf-LC	GlC	
Cap (pF)	20.2±2.0	14.7±0.9	10.8±0.9	s-GoC>sf-LC≈GlC
Rin (Mohm)	273±21	355±9	292±21	s-GoC≈sf-LC≈GlC
AP half-width (ms)	0.75±0.03	0.82±0.05	0.80±0.06	s-GoC≈sf-LC≈GlC
Sag amplitude (mV)	6.5±0.8	6.7±0.9	12.8±2.4	s-GoC≈sf-LC<GlC
AHP (mV)	−69.5±1.3	−68.3±1.2	−62.6±2.2	s-GoC≈sf-LC≈GlC
RMP (mV)	−61.7±1.8	−58.1±1.4	−56.7±1.8	s-GoC≈sf-LC≈GlC

S-GoC: small Golgi cell; sf-LC: small fusiform Lugaro cell; GlC: globular cell; Cap: whole-cell capacitance; Rin: input resistance; AP: action potential; AHP: after-hyperpolarization; RMP: resting membrane potential. S-GoC: *n* = 13, sf-LC: *n* = 8, GlC: *n* = 10. Statistical significance was examined using one-way ANOVA with Tukey's post test. > or < indicates statistically significant differences; ≈ indicates no significant difference.

### Excitatory synaptic inputs onto globular cells

**Figure 4:**
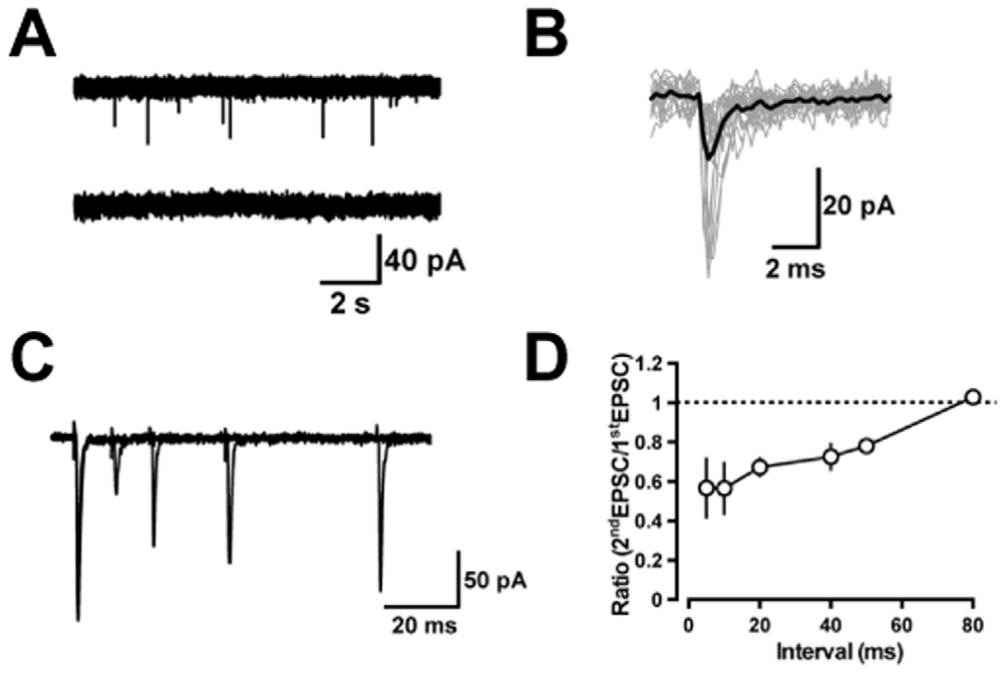
EPSCs recorded from cerebellar globular cells. Globular cells were voltage-clamped at −70 mV in the presence of 20 µM SR95531 and 1 µM strychnine. (*A*) (upper traces) example of sEPSCs recorded from a globular cell. (lower traces) a nonselective ionotropic glutamate receptor antagonist, kynurenic acid, completely blocked sEPSCs. (*B*) Individual sEPSCs (20 gray traces) were aligned at their rise time and superimposed. The averaged trace is shown by a black curve. (*C*) EPSCs evoked by double shock granular layer stimulation with changing intervals (10, 20, 40, and 80 ms) are superposed. (*D*) Ratio of the second responses to the first ones is plotted as a function of the interpulse interval (*n* = 3−5).

We examined the characteristics of excitatory synaptic inputs onto globular cells. Spontaneous EPSCs (sEPSCs) were recorded from the globular cells voltage-clamped at −70 mV in the presence of 20 µM SR95531 and 1 µM strychnine. The mean frequency and peak amplitude of sEPSCs were 2.0±0.6 Hz and 16.2±1.8 pA, respectively (*n* = 4; Fig. 4*A* upper). The excitatory current responses in globular cells are glutamatergic, because they were blocked by perfusion of kynurenic acid (1 mM) (*n* = 3, Fig. 4*A* lower). The sEPSCs (10–20 traces) recorded from each globular cell were aligned at their rise onset to obtain averaged traces (Fig. 3*B*). The rises and decays of the averaged sEPSCs were fitted by a single exponential function, respectively. The mean rise time was 0.35±0.03 ms, and the mean decay time was 1.7±0.5 ms (*n* = 4). Bath-application of 5-HT (10 µM) had no effect on either frequency (from 1.6±0.4 to 1.7±0.4 Hz, P = 0.27, n = 3) or amplitude (from 15.0±0.4 to 16.9±1.1 pA, P = 0.21, n = 3) of sEPSCs, whereas 5-HT caused excitatory inward currents in globular cells (16.0±3.6 pA, n = 3), meaning that 5-HT could directly excite globular cells. We then located the source of excitatory inputs to globular cells by extracellular electrical stimulation through a glass-stimulating electrode. We could not observe any EPSCs (peak amplitude of >10 pA) by stimulation of the molecular layer (*n* = 4). However, stimulation of the granular layers evoked EPSCs (peak amplitude of >10 pA), which showed paired-pulse depression (*n* = 5, Fig. 4*C* and *D*) rather than paired-pulse facilitation, suggesting that globular cells receive excitatory inputs through mossy fibers, like Golgi cells [27].

### Properties of IPSCs evoked in globular cells

**Figure 5:**
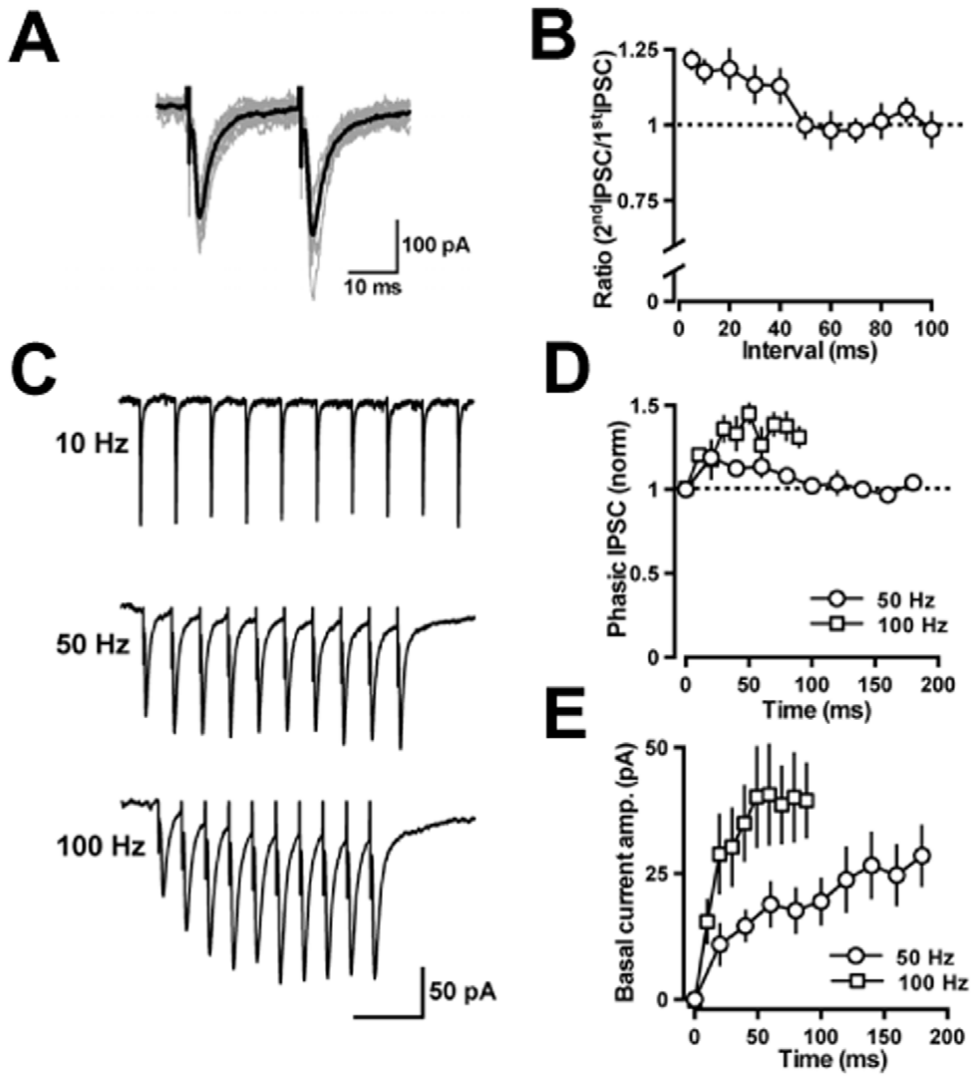
IPSCs in globular cells evoked by extracellular stimulation. (*A*) Paired pulse facilitation of IPSCs evoked in globular cells. Raw traces (gray, *n* = 12) and an averaged trace (black) of the IPSCs. (*B*) The paired-pulse facilitation of IPSCs in globular cells is plotted as a function of the interpulse interval (*n* = 3−7). (*C*) Frequency-dependent changes of evoked IPSCs. Twelve sweeps were averaged for each trace. The time scale bar indicates 200 ms (upper), 50 ms (middle), and 30 ms (lower). Stimulus artifact traces were eliminated at the basal current level. (*D*) The mean amplitudes of phasic IPSCs were measured relative to the current level preceding each stimulus artifact and normalized to the first IPSC amplitude of each train (50 Hz, *n* = 5; 100 Hz, *n* = 4). (*E*) The mean amplitudes of the basal current shift were measured immediately preceding each stimulus artifact.

We examined IPSCs evoked in globular cells by applying pulses through a glass-stimulating electrode placed at the lower border of the Purkinje cell layer close to the recorded globular cells. Paired-pulse stimulation evoked two successive IPSCs, as shown in Fig. 5A. The second IPSCs exhibited clear facilitation when the interpulse interval was <50 ms (the paired-pulse ratio, 1.18±0.04 at a 10 ms interval, *n* = 7; Fig. 5*B*). Figure 5*C* illustrates the stimulus-frequency-dependence of the evoked IPSCs produced by 10 stimuli at 10, 50, and 100 Hz. These phasic IPSCs gradually increased in the amplitude at 100 Hz (at the sixth stimulus, 145±7% of the first response, *n* = 4), but the increment was transient at 50 Hz (at the second stimulus, 119±6% of the first response, *n* = 5) (Fig. 5*D*). Slow component of the GABAergic currents increased continuously at both 50 Hz (at the last response, 30±6 pA, *n* = 5) and 100 Hz (42±8 pA, *n* = 4) (Fig. 5*E*). The paired-pulse facilitation and accumulation of the slow component are similar to those recorded at Purkinje cell-Purkinje cell synapses of 3-week-old mice [28], suggesting that axon collaterals of Purkinje cells could be involved in inhibitory synaptic transmission onto globular cells.

### Inhibitory synaptic contacts of Purkinje cells with globular cells

**Figure 6:**
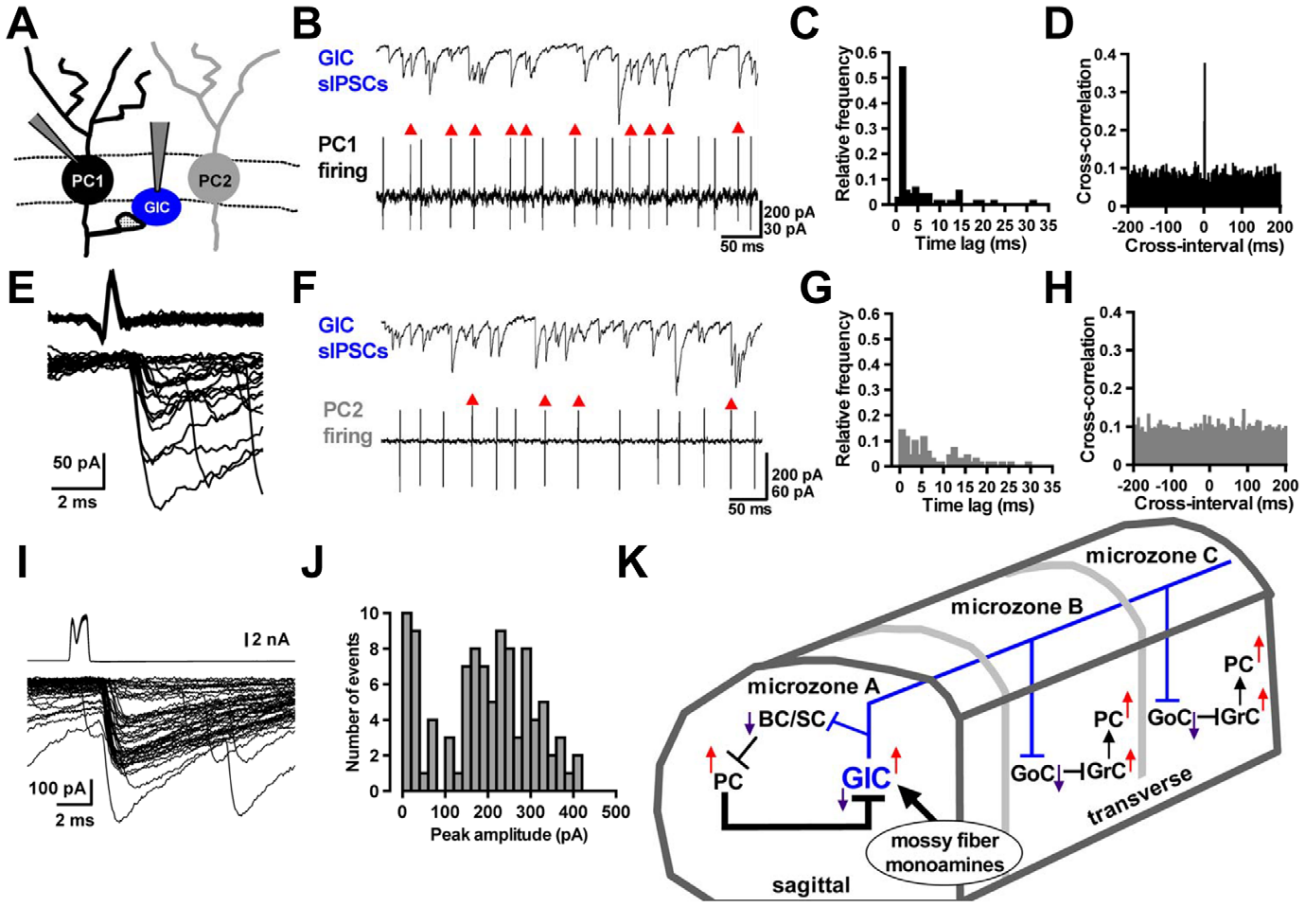
Inhibitory synaptic connections between Purkinje cells and globular cells, and a globular cell-incorporated microcircuit. (*A*) Arrangement for paired recordings from a globular cell (GlC) and a Purkinje cell (PC) (PC1 or PC2). (*B*) Simple spike discharges of PC1 (lower) and sIPSCs in the GlC (upper). Action potentials indicated by red arrowheads caused IPSCs within a 2-ms delay following each action potential. (*C*) Distributions of time lags between peaks of action potentials in PC1 and onset of IPSCs in the GlC of (*B*). (*D*) Cross-correlogram of times of spike-peak and of sIPSC-onset recorded from the same pair in (*B*). (*E*) Superimposed traces of the action potentials in the presynaptic PC1 (upper) and individual IPSCs in the GlC (lower). Twenty traces were aligned with respect to the time course of the onset of the presynaptic action potentials. Six spikes failed to evoke IPSCs. (*F*) A few action potentials of PC2 (lower) caused sIPSCs in the GlC (upper), as shown by red arrowheads. (*G*) Distributions of time lags obtained in paired recordings from PC2 and the GlC of (*F*). (*H*) Cross-correlogram of times obtained from paired recordings in (*F*). (*I*) Paired whole-cell recordings from a PC and a GlC. Depolarizing stimulation (−65 to +10 mV, 1-ms duration) was applied to the presynaptic PC with a 2-sec interval. Superimposed fifty traces of presynaptic whole cell currents in the PC (upper) and IPSCs in the GlC (lower) are shown, respectively. (*J*) The amplitude histogram was obtained from 100 evoked IPSCs recorded in the same pair of (*I*). (*K*) A schematic representation of the microcircuit with GlC predicted by the previous anatomical [6] and present studies: the PC-GlC functional connection (thick line) is indicated in the local circuit of cerebellar cortex. BC: basket cell, SC: stellate cell, GoC: Golgi cell, GrC: granule cell.

To further confirm the possibility that Purkinje cells make inhibitory synaptic contact with globular cells by electrophysiological techniques, we applied whole-cell recordings to globular cells (*n* = 9), and cell-attached recordings to Purkinje cells which are located within ∼100 µm to the recorded globular cells (23 pairs; Fig. 6*A*). In five out of the 23 paired recordings, a strong temporal correlation was recognized between spikes of a Purkinje cell and sIPSCs of a globular cell, as shown in Fig. 6*B*. In one half of the paired events, the time lag between the onsets of spikes (measured at their negative peaks) and sIPSCs (measured at the foot of rise) was less than 2 ms (Fig. 6*C*), suggesting that Purkinje cells could make direct synaptic contacts with globular cells. These observations were verified by cross-correlation analysis. The Purkinje cell-globular cell pair indicated a strong peak within 2 ms of the cross-interval (Fig. 6*D*). Twenty events of the Purkinje cell-globular cell paired recording were superimposed (Fig. 6*E*). Another pair showed a low temporal correlation between Purkinje cell spikes and sIPSCs of a globular cell (Fig. 6*F* and *G*) without any discernible peak in the cross-correlogram (Fig. 6*H*), suggesting that there is virtually no synaptic connection in this pair. Finally, we performed paired whole-cell recordings from a Purkinje cell and a globular cell. We succeeded in obtaining recordings from two connected pairs, and one of the results is shown in Fig. 6*I* and *J*. Pulse-induced Purkinje cell depolarization consistently elicited IPSCs in the globular cell (Fig. 6*I*). The averaged amplitude and coefficient variation of the 50 events were 192 pA and 0.615, respectively, including failure events (4 current responses had amplitudes of <10 pA and were classified as failures). The frequency of distribution for peak amplitudes of 100 traces is shown in Fig. 6*J*. These results indicate that sIPSCs in globular cells are induced monosynaptically by action potentials in Purkinje cells through their axon collateral synapses, consistent with the results of previous morphological studies that Purkinje cell axon collaterals may make synaptic contact with Lugaro cells and globular cells [5], [6].

## Discussion

We compared the electrophysiological properties among the three types of small-sized inhibitory interneurons (small Golgi cells, small fusiform Lugaro cells, and globular cells), which are located underneath the Purkinje cell layer in slices obtained from GAD67^+/GFP^ mice. The conspicuous feature of globular cells revealed in the present study is that they show sensitivity to both 5-HT and NA, and receive excitatory synaptic inputs through mossy fibers as well as robust inhibitory synaptic inputs through axon collaterals of Purkinje cells. Such a monosynaptic coupling between Purkinje cells and globular cells constitutes a feedback loop for globular cells, which are driven by the mossy fiber-mediated excitatory inputs under monoaminergic modulation (Fig. 6*K*, thick line). Recently, morphological properties and spontaneous spike discharge patterns of cerebellar interneurons have been analyzed by *in vivo* juxtacellular or intracellular techniques [29]–[32]. However, the small numbers of small fusiform Lugaro cells and globular cells have prevented *in vivo* studies of their physiological features. Our results indicate distinct patterns of inhibitory synaptic activity in the three types of small inhibitory interneurons: globular cells displayed sIPSCs with the highest frequency (50–60 Hz) and largest amplitude, whereas small Golgi cells displayed less frequent (up to 3 Hz) and the smallest amplitude sIPSCs, and small fusiform Lugaro cells displayed medium (∼30 Hz) sIPSCs. These physiological characteristics will be useful for classification of the small inhibitory interneurons.

### Anatomical identification of globular cells

Cytochemical observations using mice expressing GFP-labeled glycinergic and GABAergic neurons have shown that cells with globular somata are glycinergic/GABAergic and lack mGluR2 or neurogranin [6]. The globular cells which we identified in the present study are equivalent to those described in the previous paper [6]: (*i*) globular cells were located closely beneath the Purkinje cell layer and had a globular (oval) cell body of the smaller-size than other cells (Table 1); (*ii*) an injection of Alexa Fluor 594 into globular cells showed several extended processes, a few of which reached the molecular layer (Table 1); (*iii*) the percentage of globular cells to whole granular layer inhibitory interneurons was much lower than that of Golgi cells but similar to that of small fusiform Lugaro cells near the Purkinje cell layer; and (*iv*) globular cells and small fusiform Lugaro cells examined in our studies appeared to be calretinin-positive GABAergic neurons (1) [5], [17], [18].

In Fig. 1, we randomly selected small inhibitory interneurons underneath the Purkinje cell layer. Figure 1D shows a pie diagram, which indicates smaller populations of small fusiform Lugaro cells and globular cells than that of small Golgi cells. The population ratio is different from that reported in the previous morphological study [6]. This discrepancy may be explained by the difference of preparations, brain tissues fixed by perfusion for immunohistochemistry, and brain slices for electrophysiology. We suspect that that small fusiform Lugaro cells and globular cells in the near surface of the cerebellar slices seem to be damaged easily when their long axons and dendrites are cut during slice-cutting.

### Monosynaptic coupling from Purkinje cells to globular cells

The present study revealed that globular cells receive strong inhibition by Purkinje cell axon collateral inputs in the adolescent mouse cerebellum. This result is consistent with the anatomical study showing that the axon collaterals of Purkinje cells make synaptic contacts with Lugaro cells and globular cells in the adult cerebellum [5], [6]. When estimated from the averaged frequency of sIPSCs in globular cells (∼60 Hz), the averaged firing rate of Purkinje cells (∼24 Hz), and the failure rate (∼40%) observed in our Purkinje cell-globular cell paired recordings (Fig. 6*A*–*E*), a single globular cell appears to be innervated by the axon collaterals of approximately 6 Purkinje cells.

### Monoaminergic and mossy fiber excitatory inputs to globular cells

The observation that mouse cerebellar small fusiform Lugaro cells or globular cells were devoid of spontaneous firing is consistent with the property of rat Lugaro cells in the previous study [17]. Lugaro cells have been previously proposed as the primary target of 5-HT inputs in the rat cerebellar cortex, because 5-HT selectively activated Lugaro cells and did not excite basket cells/stellate cells or Golgi cells [17]. In our study, 5-HT unexpectedly excited not only small fusiform Lugaro cells but also globular cells and small Golgi cells with marked increases in their firing. 5-HT induced excitatory inward currents in globular cells without changing excitatory synaptic inputs, suggesting that 5-HT could directly excite globular cells similar to rat Lugaro cells [17]. Therefore, the sensitivity to 5-HT could not classify these neurons in mice. Another monoamine, NA, had a facilitatory effect on firing in small Golgi cells and globular cells, but not in small fusiform Lugaro cells.

Another candidate for excitatory inputs to globular cells is the mossy fibers, because electrical stimulation in the granular layer could produce EPSCs in globular cells with paired-pulse depression only at the short interpulse intervals (<100 ms) (Fig. 4*C* and *D*). These findings are similar to those described at synaptic connections of mossy fiber-Golgi cells or mossy fiber-granule cells [27], [33], and are in contrast to those at climbing fiber-Purkinje cell synaptic connection [34]. Although a previous morphological study suggests a possibility that the parallel fibers also contact with globular cells [6], the parallel fiber inputs to globular cells appear to be small, because electrical stimulation given in the molecular layer could not produce any large EPSCs in globular cells.

### Possible physiological functions of globular cells

In contrast to sagittal axonal plexus of basket cells/stellate cells, globular cells also extend long axons transversally [5]–[7], like Lugaro cells [10], [16]–[18], [35]. A plausible neuronal circuit, that includes globular cells, is illustrated in Fig. 6*K*. In this circuit, a globular cell makes synapses preferentially with basket cells/stellate cells in the sagittal axonal plexus and Golgi cells in the transversal axonal plexus [5]–[7]. Activation of globular cells by excitatory mossy fiber or monoaminergic inputs, therefore, leads to enhanced Purkinje cell activity through two distinct pathways, i.e., disinhibition through suppression of basket cells/stellate cells by globular cell outputs in the sagittal direction, and facilitation of granule cell activity through Golgi cell inhibition by globular cell outputs in the transversal direction. Subsequently, the Purkinje cell-globular cell feedback loop resumes and could silence globular cells (Fig. S2). Through globular cell outputs mediated by long transversal axons, firing of globular cells could synchronize activity of Purkinje cell clusters in different microzones (Fig. 6K) likely to contribute to motor coordination.

A previous *in vivo* study reported that 5-HT elicits one of three changes of Purkinje cell firing, i.e., inhibition, biphasic response, and excitation, correlating with the basal firing rate of Purkinje cells [36]. This correlation can be explained by the circuit shown in Fig. 6*K*. When Purkinje cells fire at high frequencies, globular cells are so strongly depressed that they could fire only a few spikes even in the presence of 5-HT, and the firing of Purkinje cells is no longer facilitated by 5-HT, but rather in some cases reduced by the direct effects of 5-HT on the Purkinje cells [37], [38]. By contrast, when Purkinje cells fire at low frequencies, 5-HT can induce robust firing of globular cells, which leads to facilitation of Purkinje cell firing. Such a rectifying action of 5-HT mediated through the globular cell-Purkinje cell circuit may modulate motor learning or coordination.

A previous behavioral study has reported that pharmacological depletion of 5-HT inputs impairs the horizontal vestibular-ocular reflex adaptation in rabbits [39]. Clinical studies have shown that timing of conditioned eyeblink responses is impaired in patients with attention-deficit/hyperactivity disorder (ADHD), some of whom show abnormal activity in 5-HT systems [40], [41]. Additionally, 5-HT precursor therapy improves cerebellar ataxia [42], [43]. The findings in this study may provide a clue to understand such modulatory actions of 5-HT on cerebellar motor functions.

## Materials and Methods

### Animals

The generation of GAD67-GFP (Δneo) mice has been described previously [20], and these heterozygous mice, used in the present study, were termed GAD67^+/GFP^ mice. The mice were maintained with a genetic background of C57BL/6 at our animal facility. All experimental procedures were approved by the RIKEN Animal Research Committee on the care and use of animals in experiments (Approval ID: H22-2-225).

### Electrophysiology

Cerebellar slices from GAD67^+/GFP^ mice aged 18–25 days were made as described previously [44]. The GAD67^+/GFP^ mice were deeply anesthetized with halothane, and sagittal slices of 230-µm thickness of cerebellar vermis were cut using a vibrating microtome (VT1000S; Leica) in an ice-cold extracellular solution containing (in mM) 252 sucrose, 3.35 KCl, 21 NaHCO_3_, 0.6 NaH_2_PO_4_, 9.9 glucose, 1 CaCl_2_, and 3 MgCl_2_ and gassed with a mixture of 95% O_2_ and 5% CO_2_ (pH 7.4). The slices were maintained at 30°C for 30 min in a holding chamber, where they were submerged in artificial cerebrospinal fluid (ACSF) containing (in mM) 138.6 NaCl, 3.35 KCl, 21 NaHCO_3_, 0.6 NaH_2_PO_4_, 9.9 glucose, 2 CaCl_2_, and 1 MgCl_2_ (bubbled with 95% O_2_ and 5% CO_2_ to maintain the pH at 7.4). Thereafter, slices were kept at room temperature.

Individual slices were transferred to a recording chamber attached to the stage of a microscope (BX51WI, Olympus) and superfused with oxygenated ACSF. Small inhibitory interneurons underneath the Purkinje cell layer were visually identified with GFP fluorescence in the cerebellar cortex of GAD67^+/GFP^ mice. Recordings were performed from neurons of lobules III–IX to limit the variability depending on the specialization of different regions of the cerebellar cortex [17]. Extracellular spike activity in neurons was observed by loose cell-attached voltage-clamp at holding potential of 0 mV. Glass electrodes used for the cell-attached recordings had resistances of 3–4 MΩ when filled with ACSF. To isolate IPSCs, patch pipettes (2–4 MΩ) were filled with an intracellular solution containing (in mM) 140 CsCl, 0.1 CaCl_2_ 1 K-EGTA, 10 Na-HEPES, 3 Mg-ATP, and 0.4 Na-GTP (pH 7.4), and the holding potential was set at −70 mV to detect IPSCs as larger inward current responses. A nonselective ionotropic glutamate receptor antagonist, kynurenic acid (1 mM) (or 10 µM 2,3-dioxo-6-nitro-1,2,3,4-tetrahydrobenzo[*f*]quinoxaline-7-sulfonamide disodium salt: NBQX and 30 µM dl-2-amino-5-phosphonovaleric acid: APV), was added to the ACSF throughout the IPSC recordings. Whole-cell capacitance was calculated by integrating the area under the transient following a 10 mV hyperpolarizing voltage step from the holding potential, −70 mV. Miniature IPSCs were recorded using a CsCl-based internal solution in the presence of TTX (0.5 µM). To observe evoked synaptic responses, focal stimulation (10–20 V, 0.1–0.2 ms) was applied by a grass microelectrode (tip diameter of 1–2 µm) containing ACSF. To isolate EPSCs, interneurons were voltage-clamped at −70 mV in the presence of 20 µM SR95531 and 1 µM strychnine. Series resistance (10–18 MΩ) was compensated by 70% and monitored using 2 mV hyperpolarizing voltage steps, and experiments were discarded if the value changed by ∼20%. Unless otherwise noted, most experiments were performed at room temperature (24–26°C). SR95531 was obtained from Tocris Cookson, and TTX was from Wako. All other chemicals were from Sigma.

The firing and membrane properties of small inhibitory interneurons (Fig. 3 and Table 2) were examined by whole-cell current-clamp recordings with patch pipettes (3–5 MΩ) filled with a K^+^-based intracellular solution containing (in mM) 75 KCH_3_SO_3_, 70 KCl, 0.1 CaCl_2_ 1 K-EGTA, 10 Na-HEPES, 3 Mg-ATP, and 0.4 Na-GTP (pH 7.4). We used an internal solution containing 70 KCl because we easily observed spontaneous postsynaptic currents as one of the criteria for the distinction of small inhibitory interneurons. The electrophysiological properties listed in Table 2 were observed immediately after transition into the whole-cell configuration. The input resistance was measured from a steady-state voltage level during a 400 ms-hyperpolarizing current (100 pA) injection. The action potential half-width was calculated as the width of the action potential, measured at a point half-way between the threshold and the action potential peak. The action potential threshold was determined as the first point in the voltage trajectory in which the slope exceeded 20 V/s [45]. The voltage sag was the difference between the minimum membrane potential and steady-state level immediately before the offset of the hyperpolarizing current (−100 pA, 400 ms-duration). After hyperpolarization (AHP) value was the minimum membrane potential after the offset of the depolarizing current injection (+200 pA, 400 ms-duration).

Paired whole-cell recordings were performed from a Purkinje cell and a globular cell. Patch electrodes used for the whole-cell voltage-clamp recordings from presynaptic Purkinje cells had resistance of 2–3 MΩ when filled with the K^+^-based intracellular solution described before. In Fig. 6*I*, presynaptic whole-cell recordings were obtained from a Purkinje cell located on the apical side 150 µm from a postsynaptic globular cell recorded.

The membrane potentials and currents were recorded using the amplifier MultiClamp 700B (Molecular Devices) and pCLAMP 9.2 software (Molecular Devices), digitized, and stored on a computer disk for off-line analysis. All signals were filtered at 2–4 kHz and sampled at 5–20 kHz, and synaptic events were analyzed with a threshold of 10 pA. The frequencies of synaptic events are shown by the number of synaptic events (for 30 sec) divided by the time duration. Spike firing and synaptic events were analyzed using Mini analysis program 6.0 (Synaptosoft), pCLAMP 9.2 software, and Kyplot software 5.0 (Kyence). All data are expressed as the mean ± standard error of the mean (s.e.m.). The number in parentheses represents the number of tested neurons. Unless otherwise stated, the level of significance was determined by the paired Student's *t*-test between groups.

### Morphological analyses

It was difficult to unequivocally distinguish the three types of small inhibitory interneurons under Nomarski optics. To visualize their shape, 40 µM Alexa Fluor 594 (Invitrogen) was added to the internal solution. After whole-cell patch experiments, the patch pipette was removed from the cell. Fluorescent images were obtained by fluorescent microscopy (BX51WI, Olympus) with a water immersion objective lens (20×, NA 0.50, Olympus). Images were acquired using a digital camera (ORCA-ER, Hamamatsu Photonics). Z-stacked images were generated from 3–5 manually captured sections with thickness of 5–8 µm and analyzed using image software, Aqua-Lite 1.2 (Hamamatsu Photonics). Images of small inhibitory interneurons infused with the fluorescent dye were accumulated through the whole experiments. In order to carry out detailed analyses of the morphological properties as shown in Table 1, we selected the best-preserved cell images. The small axis (SA) of soma is the vertical diameter, and the great axis (GA) of soma is the horizontal diameter parallel to the PC layer. The deformation index of soma is expressed as GA/SA.

### Immunohistochemistry

GAD67^+/GFP^ mice (PND21–30) were anesthetized and perfused intracardially with 4% paraformaldehyde. Their brains were excised, postfixed and equilibrated with 30% sucrose in physiological buffered saline. Frozen sections were prepared at 20 µm thickness using a cryostat microtome (CM3050S, Leica) and mounted on MAS-coated glass slides. The sections were incubated overnight with a rabbit polyclonal antibody against calretinin (1∶2000, Swant, 7699/4) at 4°C and then stained with Alexa Fluor 594-conjugated anti-rabbit antiserum (1∶500, Invitrogen, A-11037) for 1 h at room temperature. Observation was made using a microscope (BX61, Olympus) and a confocal laser scanning microscope (FV1000D, Olympus). Images were generated by stacks of 7–10 captured sections spaced by 1.67 µm.

## Supporting Information

Figure S1 **Calretinin-positive interneurons underneath the Purkinje cell layer have similar distribution of the deformation index to globular cells and small fusiform Lugaro cells.** (*A*) Immunoreactivity for anti-calretinin antibody in the cerebellar cortex of a GAD67^+/GFP^ mouse. Left: GFP fluorescence of the GAD67^+/GFP^ mouse. Middle: An oval cell body was stained by the anti-calretinin antibody as indicated by an arrow. Right: A merged image of the left and middle images. (*B*) High magnification of the calretinin-positive cell body in (*A*). Scale bars: (*A*) 20 µm; (*B*) 10 µm. (*C*) Deformation indexes (GA/SA) for the soma of calretinin-positive (CR+: red dots) and calretinin-negative (CR−: black dots) GABAergic interneurons located underneath the Purkinje cell layer are plotted versus GA. The straight line is the least-squares fit to red dots: GA/SA = 0.092×GA+0.56. GA: great axis of soma (µm), SA: small axis of soma (µm). (*D*) Deformation indexes (GA/SA) for the soma of whole-cell recorded small inhibitory interneurons are plotted versus GA. Black: small Golgi cells (s-GoCs), Blue: globular cells (GlCs), Green: small fusiform Lugaro cells (sf-LCs). The straight line is the least-squares fit to a data set of GlCs and sf-LCs: GA/SA = 0.076×GA+0.52. The distribution of the deformation indices for CR+ (*C*) and a group of GlCs and sf-LCs (*D*) seems similar.
(TIF)

Figure S2 **Effects of blocking inhibitory synaptic transmission on 5-HT-induced excitation in globular cells.** Under control conditions, application of 5-HT (10 µM) elicited action potentials in 5 of 8 globular cells. Although blocking inhibitory synaptic transmission onto globular cells by perfusion of SR95531 (20 µM) and CGP55845 (2 µM) failed to elicit firing of globular cells, the treatment significantly enhanced and prolonged the 5-HT-induced firing of globular cells (2.1±0.8 Hz, *n* = 8 versus 17.5±4.1 Hz, *n* = 5; Mann-Whitney *U* test, P<0.001).
(TIF)
