# Supplementary figures and images for: Cerebellar Globular Cells Receive Monoaminergic Excitation and Monosynaptic Inhibition from Purkinje Cells
Source: PLoS One. 2012 Jan 3;7(1):e29663. doi: 10.1371/journal.pone.0029663 (PMC3250469; doi:10.1371/journal.pone.0029663)

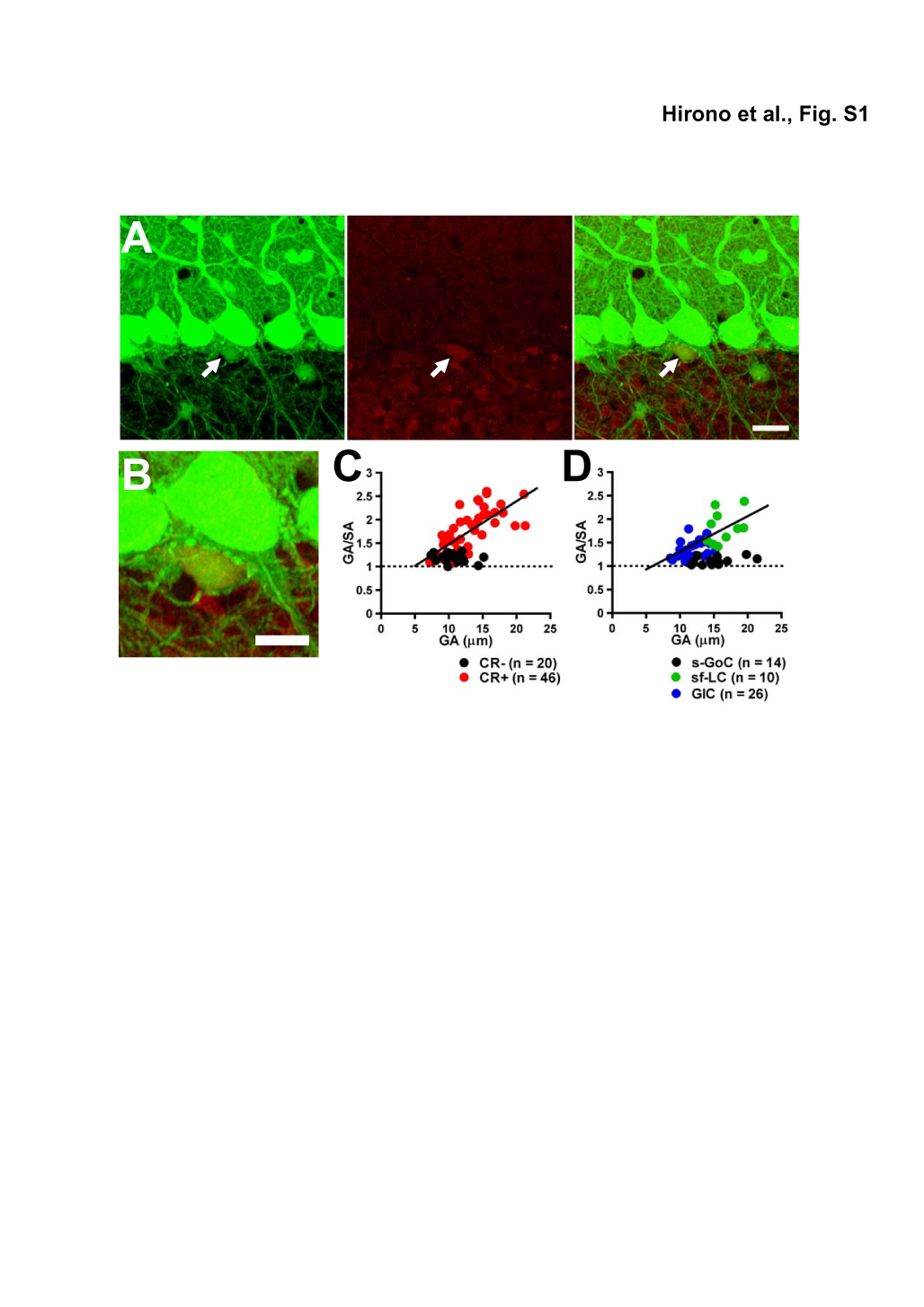

Supplement: Figure S1 — Calretinin-positive interneurons underneath the Purkinje cell layer have similar distribution of the deformation index to globular cells and small fusiform Lugaro cells. (A) Immunoreactivity for anti-calretinin antibody in the cerebellar cortex of a GAD67+/GFP mouse. Left: GFP fluorescence of the GAD67+/GFP mouse. Middle: An oval cell body was stained by the anti-calretinin antibody as indicated by an arrow. Right: A merged image of the left and middle images. (B) High magnification of the calretinin-positive cell body in (A). Scale bars: (A) 20 µm; (B) 10 µm. (C) Deformation indexes (GA/SA) for the soma of calretinin-positive (CR+: red dots) and calretinin-negative (CR−: black dots) GABAergic interneurons located underneath the Purkinje cell layer are plotted versus GA. The straight line is the least-squares fit to red dots: GA/SA = 0.092×GA+0.56. GA: great axis of soma (µm), SA: small axis of soma (µm). (D) Deformation indexes (GA/SA) for the soma of whole-cell recorded small inhibitory interneurons are plotted versus GA. Black: small Golgi cells (s-GoCs), Blue: globular cells (GlCs), Green: small fusiform Lugaro cells (sf-LCs). The straight line is the least-squares fit to a data set of GlCs and sf-LCs: GA/SA = 0.076×GA+0.52. The distribution of the deformation indices for CR+ (C) and a group of GlCs and sf-LCs (D) seems similar. (TIF) [file pone.0029663.s001.tif]

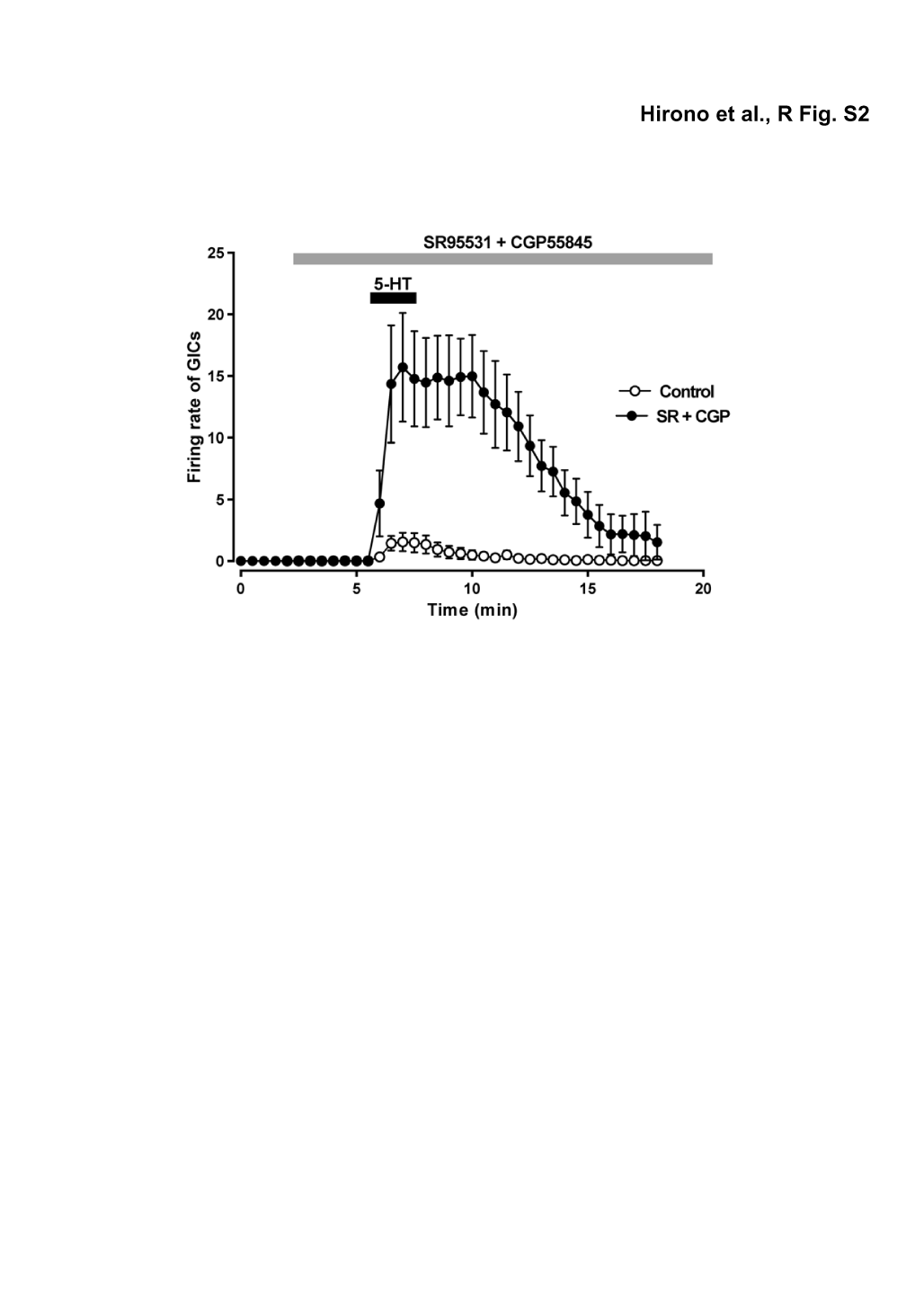

Supplement: Figure S2 — Effects of blocking inhibitory synaptic transmission on 5-HT-induced excitation in globular cells. Under control conditions, application of 5-HT (10 µM) elicited action potentials in 5 of 8 globular cells. Although blocking inhibitory synaptic transmission onto globular cells by perfusion of SR95531 (20 µM) and CGP55845 (2 µM) failed to elicit firing of globular cells, the treatment significantly enhanced and prolonged the 5-HT-induced firing of globular cells (2.1±0.8 Hz, n = 8 versus 17.5±4.1 Hz, n = 5; Mann-Whitney U test, P<0.001). (TIF) [file pone.0029663.s002.tif]
